# A probabilistic multi objective decision framework for reactive power planning in der integrated microgrids enhancing sustainability and resilience in the egyptian grid

**DOI:** 10.1038/s41598-026-66944-w

**Published:** 2026-08-20

**Authors:** Rasha Elazab, Eman Kamal Sakr, Maged Abo‑Adma, Abdallah Mohammed

**Affiliations:** Faculty of Engineering, Department of Electrical Power and Machines Engineering, Helwan University, Cairo, Egypt

**Keywords:** Distributed energy resources (DERs), Multi-objective optimization, Microgrid planning, Sustainable energy, Egyptian grid, Life-cycle assessment emission, Carbon footprint, Energy science and technology, Engineering

## Abstract

The integration of high-penetration Distributed Energy Resources (DERs) into conventional grids is pivotal for de-carbonization but introduces significant stability challenges. This study addresses voltage violations and feeder congestion in Egyptian distribution networks using a probabilistic four-dimensional multi-objective optimization framework that supports Sustainable Development Goals (SDGs) 7 (Affordable and Clean Energy) and 13 (Climate Action). The methodology combines the Two-Point Estimation Method (2PEM) for uncertainty quantification with the NSGA-II algorithm to optimize technical, economic, environmental, and social objectives for an IEEE 33-bus test system. Results demonstrate that optimal DER sizing alone is insufficient and that dedicated reactive power planning is essential. Among five evaluated planning strategies, a hybrid configuration of inverters and D-STATCOMs delivers superior performance. This optimal scenario achieves the best voltage profile (0.975 p.u.), the highest annual loss savings ($61,900), and creates 52 per MW at full-time equivalent jobs, thereby advancing SDG 8 (Decent Work and Economic Growth) while reducing grid dependency to 22%. Although it requires a higher initial investment, its techno-economic and social benefits justify adoption for strengthening modern grids. The study provides a critical decision-support framework for policymakers to enhance the resilience and sustainability of renewable-integrated power systems in Egypt and similar emerging economies.

**Supplementary Information:**

The online version contains supplementary material available at 10.1038/s41598-026-66944-w.

## Introduction

The ongoing transition toward decentralized smart grids is intensifying the integration of Distributed Energy Resources (DERs) such as photovoltaic (PV), wind, and hybrid systems to meet de-carbonization and energy diversification goals^1–3^. Egypt's Integrated Sustainable Energy Strategy (ISES) 2035 aims to achieve 42% renewable energy penetration, supported by national frameworks that promote grid flexibility and reliability^4,5^. However, high DER penetration in traditional radial systems, such as the IEEE 33-bus feeder, introduces voltage deviations, feeder congestion, and thermal overloading under uncertainty^6,7^. The stochastic nature of renewable generation necessitates probabilistic analysis methods, such as the Two-Point Estimation Method (2PEM), for realistic operational planning^8–10^. Recent reports on Egyptian distribution networks document recurrent voltage violations, feeder congestion, and overloading under increasing demand and renewable integration, particularly in heavily loaded urban and coastal regions. These operational challenges highlight the need for advanced probabilistic reactive power planning frameworks that can ensure voltage stability and reliable operation under worst-case loading and renewable uncertainty. DER integration planning is inherently a multi-objective optimization (MOO) problem that requires balancing techno-economic objectives^11^. Advanced algorithms such as NSGA-II effectively generate Pareto-optimal solutions for such conflicting criteria^12–14^. Nevertheless, reactive power support remains a persistent challenge in modern distribution systems with high DER penetration^15^. Reactive power is vital for maintaining voltage stability, reducing feeder losses, and preventing voltage collapse under variable renewable generation. Although IEEE 1547 mandates reactive capability for inverter-based DERs, practical adoption remains limited due to coordination complexity and cost. D-STATCOMs offer fast, flexible reactive compensation^16^, while multi-functional inverters can also support voltage regulation^17^. However, most studies treat these devices independently, neglecting their interaction or system-level optimization under uncertainty^18,19^. In this study, the advantage of D-STATCOM-based compensation is quantitatively demonstrated through a direct comparison of multiple strategies under identical probabilistic conditions, showing that D-STATCOM-based scenarios achieve lower active and reactive power losses, improved worst-case voltage levels, and reduced feeder loading.

The methodological strengths and weaknesses of the studies in the literature review shown in Table 1 directly impact the validity and applicability of existing findings. Studies employing deterministic approaches^1,4,6,15–19^ may overestimate system performance under real-world variability, while those excluding environmental or social dimensions^2,6,7,15–19,24^ provide incomplete sustainability assessments. Conversely, recent studies incorporating digital twin technology^2^ and community behavior modeling^27^ offer valuable insights into real-time monitoring and social dynamics but lack integrated optimization frameworks^12^. Moreover, recent literature highlights the growing importance of sustainability indicators such as life-cycle emissions, job creation, and grid dependency in microgrid planning^21,22^ Despite this, few studies integrate these dimensions into a unified probabilistic optimization framework. Table 1 provides a comparative analysis of recent and relevant literature, highlighting methodological gaps in uncertainty modeling, reactive power sources, environmental assessment, and social metrics. This table underscores the need for a holistic approach that simultaneously addresses technical, economic, environmental, and social objectives under uncertainty In contrast to existing international studies that address reactive power planning, DER integration, or sustainability assessment as separate problems^25–27^, this work proposes a unified probabilistic four-dimensional optimization framework. Recent reviews highlight the advancement of multi-objective algorithms^28^ and the specific application of inverter-based reactive support^21^, yet they often remain from comprehensive life-cycle and social assessments. Similarly, while probabilistic life-cycle analysis is gaining traction^29^, its integration into a holistic, uncertainty-aware planning framework for reactive power is lacking. Recently, several studies have explored advanced metaheuristic and hybrid optimization algorithms to address complex nonlinear problems in modern power and energy systems. Hybrid approaches combining swarm intelligence techniques have shown improved convergence behavior and solution quality compared to conventional algorithms. For instance, hybrid metaheuristic frameworks integrating moth-flame optimization with particle swarm optimization have demonstrated superior performance in solving complex engineering optimization problems in power transmission and distribution systems^30^. Similarly, recent studies have investigated chaotic optimization strategies and enhanced swarm-based algorithms for improving global exploration and stability in distribution network optimization problems^31,33^. In addition, comprehensive reviews of metaheuristic algorithms highlight their effectiveness in solving large-scale multi-objective optimization problems due to their ability to balance exploration and exploitation while handling nonlinear and multi-dimensional search spaces^32^. These advances confirm the growing role of metaheuristic optimization techniques in addressing the increasing complexity of modern energy systems and microgrid planning problems.

**Table 1:** Literature Review and Comparative Analysis of previous Studies.

References	Uncertainty	MOO	Reactive source	Environmental (LCA)	Social	Key contribution	Methodological gap
^1,11–13^	✗	✓	✗	✗	✗	MOO methods & hybrid system design	Deterministic, ignore uncertainty & sustainability
^2^	✗	✗	✗	✗	✗	Digital twin review in smart grids	No optimization/reactive power focus
^4,18^	✗	✓	✗	✓	✓	Renewable integration policy & outlook reviews	No uncertainty/reactive power modeling
^6,15,16^	✗	✗	✓ (D-STATCOM/PV)	✗	✗	D-STATCOM applications for compensation	Deterministic, device-specific, no MOO/sustainability
^7^	✓	✓	✓ (Inverter & EV)	✗	✗	MOO with storage & EVs	Neglect environmental/social dimensions
^17^	✗	✓	✓ (Multifunction Inverter )	✓	✗	Multi-functional inverter for PQ improvement	No uncertainty modeling
^19^	✗	✓	✓ (Hybrid AC/DC)	✗	✓	Distributed control of hybrid grids	Deterministic framework
^20^	✓	✗	✗	✗	✗	Probabilistic algorithm for PV generation	No reactive power/social metrics
^21,22^	✗	✓	✗	✗	✓	Community behavior & social microgrid factors	Limited scope, no technical optimization
^12^	✗	✓	✗	✗	✗	Microgrid EMS using PHIL control	Lacks uncertainty/reactive power focus
^25^	✓	✗	✗	✗	✗	Energy system reference & probabilistic methods	No reactive power/social metrics
^27^	✗	✓	✗	✗	✓	Community behavior & social factors modeling	No environmental/technical optimization
^28^	✓	✓	✗	✗	✗	Review of MOO techniques for sustainable power systems	Focus on algorithms, lacks an integrated planning framework
^21^	✗	✓	✓ (Inverter-based)	✗	✗	Reactive power management using smart inverters	Deterministic, no environmental or social metrics
^29^	✓	✗	✗	✓	✗	Probabilistic LCA of hybrid renewable systems	No MOO, reactive power, or social dimension
^30–33^	✗	✓	✗	✗	✗	Recent developments in metaheuristic and hybrid optimization algorithms for engineering problems	Focus primarily on algorithmic development rather than integrated probabilistic microgrid planning
Proposed	✓	✓	✓ (Inverter + D-STATCOM)	✓	✓	Probabilistic 4D MOO with LCA & social metrics	**-**

The novelty of the proposed approach, therefore, lies in the simultaneous consideration of uncertainty-aware technical performance, full life-cycle environmental impact, economic feasibility, and quantified social benefits within a single NSGA-II–based decision-support model. Furthermore, the joint probabilistic optimization of inverter-based reactive capability and D-STATCOM deployment, under thermal and operational constraints, distinguishes this study from prior deterministic or device specific approaches. This integrated perspective enables more realistic and sustainable planning of DER-rich microgrids, particularly for stressed distribution networks in emerging economies.

Accordingly, the main contributions of the proposed work are as follows:

- **Development of a unified probabilistic multi-objective optimization framework** that integrates the Two-Point Estimation Method (2PEM) with the NSGA-II algorithm to simultaneously optimize technical, economic, environmental, and social objectives under renewable generation and load uncertainties.
- **Comparative assessment of five reactive compensation strategies** including inverter-based, capacitor-based, D-STATCOM-based, and hybrid Inverter + D-STATCOM configurations within the same probabilistic environment to identify the most effective techno-economic solution.
- **Incorporation of comprehensive sustainability metrics**, including Life Cycle Assessment (LCA), thermal line capacity, job creation, grid dependency, and CO₂ reduction, providing a balanced evaluation across technical, economic, environmental, and social dimensions.
- **Integration of inverter reactive capability and thermal constraints** into the optimization framework, enhancing voltage regulation, improving network reliability, and ensuring safe operation under fluctuating renewable and demand conditions.

The proposed approach differs from existing methods by jointly considering uncertainty aware technical performance, economic feasibility, full life-cycle environmental impact, and quantified social benefits within a single optimization framework. This enables decision-makers to evaluate tradeoffs and select robust solutions that are not attainable through conventional deterministic or single-objective approaches.

## Methodology

The probabilistic formulations, renewable generation models, and power flow equations adopted in this study are based on well-established models reported in the literature and are referenced accordingly. The novelty of the proposed approach lies not in the individual analytical equations, but in their unified integration within a probabilistic multi-objective optimization framework that simultaneously evaluates technical, economic, environmental, and social objectives under uncertainty.

### System configuration

**Fig. 1:**
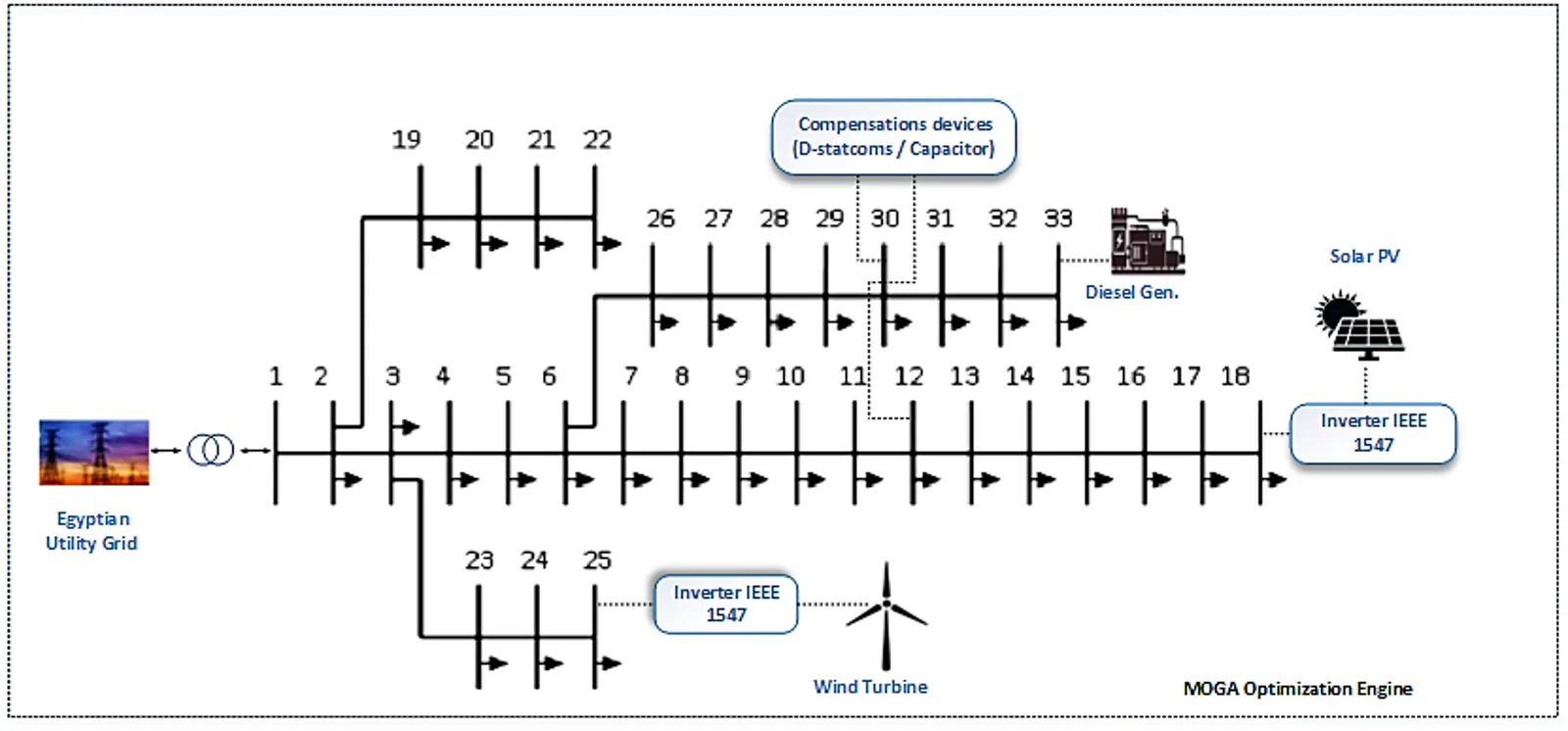
Single-line diagram of the grid-connected microgrid test system

The studied grid-connected microgrid is based on the IEEE-33 bus Egyptian distribution feeder. All DERs are IEEE 1547–2023 compliant and connected through smart inverters, the grid-connected microgrid model was implemented and simulated using MATLAB R2021a. All power flow analysis and probabilistic assessments were conducted using the Matpower 8 toolbox to solve the AC optimal power flow problems .The system topology, including DER placements and compensation devices, is illustrated in Fig. 1^17^.

### Base system configuration and benchmark metrics

The study employs the standard IEEE 33-bus radial distribution test system^6^, which has the following base characteristics:

#### System parameters

I. Total active load: 3.715 MW
II. Total reactive load: 2.300 MVAr
III. Nominal voltage: 12.66 kV
IV. Base MVA: 100 MVA
V. Total line length: 38.8 km
VI. System topology: Radial with 33 buses and 32 branches

#### Base case performance

I. Total active power losses: 185.6 kW
II. Total reactive power losses: 105.2 kVAr
III. Minimum voltage: 0.927 p.u. (at bus 18)
IV. Maximum voltage: 1.000 p.u. (at substation bus 1)
V. Maximum line loading: 148.2% (branch 17–18)
VI. Total load served: 100% from grid

#### DER integration base data

I. Solar potential (Red Sea region): Average irradiance 5.8 kWh/m^2^/day
II. Wind potential (Red Sea coast): Average speed 7.2 m/s, Weibull k = 2.1, c = 8.0
III. Load growth scenario: 115% of base load (representing Egyptian stressed feeder conditions^5,11^)

These baseline parameters and performance metrics enable direct comparison with previous studies using the IEEE 33-bus system^6,17^ and ensure reproducibility of the optimization results.

### Probabilistic framework

The operational stability of microgrids is inherently challenged by the stochastic nature of renewable generation and load demand^8,9^. To address this, a robust probabilistic framework based on the Two-Point Estimation Method (2PEM) is employed. The 2PEM efficiently quantifies uncertainty propagation by calculating the expected value and variance of system states, offering a computationally tractable alternative to Monte Carlo simulations^10^. The core 2PEM formulation is given by Eqs. (1–6) represent expected values, PV, and wind models.

#### Two-point estimation method (2PEM)^10^

1 $$E \left[Y\right]=\sum_{i=1}^{2n}\omega i Y \left({X}_{i}\right), var\left[Y\right]= \sum_{i=1}^{2n}{\omega i \left[Y \left({X}_{i}\right)-E\left[Y\right]\right]}^{2}$$

Equation (1) constitutes the core of the probabilistic framework, where Y denotes the vector of system output states of interest, such as bus voltage magnitudes and power losses, while Xi represents the uncertain input variables, including solar irradiance, wind speed, and load demand. The weighting factors ωi are determined according to the standard 2PEM procedure^10^. The resulting expected values and variances are propagated through the probabilistic load flow (Eq. 6) and subsequently used in the evaluation of the technical objective function (Eq. 15), enabling the optimization to explicitly account for uncertainty. This efficiently captures uncertainty propagation in power flow results compared to Monte Carlo simulation. The Two-Point Estimation Method offers a significant reduction in computational burden compared to Monte Carlo simulation. While the Two-Point Estimation Method efficiently captures the first- and second-order statistical moments of uncertain inputs, it primarily represents average system behavior and moderate variability. Extremely rare events such as prolonged renewable generation droughts or simultaneous peak load conditions may not be fully represented by moment-based methods. However, the main objective of this study is long-term planning evaluation of reactive power strategies rather than operational reliability analysis. For this purpose, 2PEM provides a computationally efficient probabilistic representation when embedded within the NSGA-II optimization loop. So, The Two-Point Estimation Method has been widely adopted for probabilistic power system analysis because it accurately estimates the statistical moments of system states with significantly fewer power-flow evaluations compared with Monte Carlo simulation^10^.

#### PV power output

The power output from PV systems is modeled considering solar irradiance and ambient temperature, which are key stochastic variables^8^. The output power is calculated as:

2 $${P}_{pv}= {P}_{STC}\frac{G}{{G}_{STC}} \left[ 1+ \alpha \left( {T}_{C}-{T}_{STC}\right)\right]$$

3 $${T}_{C= }{T}_{a }\left(\frac{NOCT-20}{800}\right) G$$

where NOCT is nominal operating cell temperature and Ta is ambient temperature.

#### Wind Turbine Power Model

The power output from wind turbines is a function of stochastic wind speed, typically characterized by a Weibull distribution^9^. The model is defined as:

4 $${P}_{WT}= \left\{\begin{array}{c}0 , v<{v}_{ci}\\ {P}_{r}\left(\frac{v-{v}_{ci}}{{v}_{r}-{v}_{ci}}\right) , {v}_{ci}\le v\le {v}_{r} \\ {P}_{r} ,{ v}_{r}\le v\le {v}_{co}\end{array}\right.$$

Expected wind output considering the Weibull distribution^9^:

5 $$E \left[ {P}_{WT} \right]={\int}_{0}^{\infty }P\left(v\right) \rho \left(v\right) dv , \rho \left(v\right)=\frac{k}{c} {\left(\frac{v}{c}\right)}^{k-1}{e}^{{-(\frac{v}{c})}^{k}}$$

The Weibull shape and scale parameters used in Eq. (5) were adopted based on representative meteorological data reported for the Red Sea coast, Egypt^20^. The cut-in, rated, and cut-out wind speeds of the modeled wind turbine are set to 3 m/s, 12 m/s, and 25 m/s, respectively. The resulting expected wind power output constitutes a stochastic input to the system power balance constraint.

#### Probabilistic load flow

For each scenario generated by the 2PEM, a probabilistic load flow is executed to obtain the statistical moments of bus voltages, which are critical for assessing system stability^10^ .For each 2PEM scenario, the probabilistic load flow yields bus voltage statistics^10^:

6 $$E \left[ {V}_{i}\right]= {\sum}_{k}{\omega}_{k} {V}_{i}^{k} , \sigma {V}_{i}= \sqrt{{\sum}_{k}{\omega}_{k} ({{V}_{i}^{k}-E \left[ {V}_{i}\right])}^{2}}$$

Outputs feed the optimization engine as scenario-based performance indices.

#### Life-Cycle Assessment Emissions (LCA) Modeling

To accurately capture the environmental impact, a comprehensive life-cycle assessment (LCA) model is implemented. This model accounts for emissions from manufacturing to decommissioning, providing a full picture of the carbon footprint [11, 17 and 23]. The total life-cycle emissions are calculated as: Life-cycle emissions (Eq. 7–13) include manufacturing, transportation, installation, operation, and decommissioning. Each emission stage is expressed in CO₂-equivalent terms. To account for total environmental impact, life-cycle CO₂-equivalent emissions are calculated for five stages^23^**:**

7 $${E}_{LCA}= {E}_{MFG}+ {E}_{TRN}+ {E}_{INS}+ {E}_{OPR }+ {E}_{DEC}$$

Each stage is defined as follows:

Manufacturing^23^

8 $${E}_{MFG}= \sum_{i}{ (M}_{i}X E{F}_{M,i }) $$

where ${(M}_{i}$ is the mass or rated capacity of material *i* (modules, turbines, generators).

Transportation^23^

9 $${E}_{TRN}= \sum_{j}{ (D}_{j} { W}_{j} X E{F}_{T, j })$$

With D_j_ transport distance and W_j_ component weight.

Installation and Construction^23^

10 $${E}_{INS}={P}_{rated} X E{F}_{INS}$$

Operation and Maintenance^23^

11 $${E}_{OPR}= \sum_{t=1}^{N}( {E}_{gen,t} X E {F}_{OPR })$$

Decommissioning and Recycling^23^

12 $${E}_{DEC }= {P}_{rated }X ( E{F}_{D }- {R}_{REC })$$

where ${R}_{REC}$ the recycled fractions avoided emission.

Finally, the emission intensity, which normalizes the total emissions by the energy generated, is a key performance metric the life-cycle emission intensity expressed in (ton CO₂/kWh) is^23^:

13 $${EF}_{LCA}=\frac{{E}_{LCA}}{\sum_{t=1}^{N}{E}_{gen.t}}$$

The emission factors employed in Eqs. (8) – (12), corresponding to manufacturing, transportation, installation, operation, and decommissioning stages, are derived from established life-cycle assessment databases and recent literature^23^. These parameters directly contribute to the environmental objective function defined in Eq. (17).

### Multi-objective optimization

The optimization minimizes four objectives: f_tech_, f_eco_, f_env_, and maximizes SS. Constraints enforce power balance, voltage, and DER limits. Technical, economic, environmental (LCA), and social sub-objectives are shown in Eqs. 15–18.

#### Objective vector

14 $$ \min F\left( x \right) = w_{1} f_{{{\mathrm{tech}}}} \left( x \right) + w_{2} f_{{{\mathrm{env}}}} \left( x \right) + w_{3} f_{{{\mathrm{eco}}}} \left( x \right) - w_{4} SS\left( x \right) $$

where ${\mathrm{w}}_{\mathrm{i}}$ the weighting coefficients for each criterion and SS are represents the Social Score, subtracted here to maximize social benefits.

#### Constraints

The optimization is subject to standard power system operational constraints to ensure feasibility and reliability^4,6^, including power balance, voltage limits, DER capacity bounds, and thermal limits of lines. System operation is subject to the following constraints:

- Power Balance

$$\sum {\mathrm{P}}_{\mathrm{g}}+ {\mathrm{Q}}_{\mathrm{g}}=\sum_{\mathrm{i}=1}^{\mathrm{N}}\left({\mathrm{p}}_{\mathrm{d}\mathrm{e}\mathrm{m}\mathrm{a}\mathrm{n}\mathrm{d},\mathrm{i}}+\mathrm{j} {\mathrm{Q}}_{\mathrm{d}\mathrm{e}\mathrm{m}\mathrm{a}\mathrm{n}\mathrm{d},\mathrm{i}}\right)+ {\mathrm{P}}_{\mathrm{l}\mathrm{o}\mathrm{s}\mathrm{s} }+ {Q}_{loss}$$

b) Voltage Limits

$${\mathrm{V}}_{\mathrm{m}\mathrm{i}\mathrm{n}}\le {\mathrm{V}}_{\mathrm{i}} \le {\mathrm{V}}_{\mathrm{m}\mathrm{a}\mathrm{x}}$$

c) DER Capacity Bounds

$${P}_{DER}^{min }\le {P}_{DER}\le {P}_{DER}^{max}$$

d) D-STATCOM Installation Criteria

The placement and sizing of D-STATCOMs are determined through optimization while respecting the following technical criteria.

1. Reactive power capacity limits

$${\mathrm{Q}}_{\mathrm{D}\mathrm{S}\mathrm{T}\mathrm{A}\mathrm{T}\mathrm{C}\mathrm{O}\mathrm{M},\mathrm{m}\mathrm{i}\mathrm{n}}\le {\mathrm{Q}}_{\mathrm{D}\mathrm{S}\mathrm{T}\mathrm{A}\mathrm{T}\mathrm{C}\mathrm{O}\mathrm{M} }\le {\mathrm{Q}}_{\mathrm{D}\mathrm{S}\mathrm{T}\mathrm{A}\mathrm{T}\mathrm{C}\mathrm{O}\mathrm{M},\mathrm{m}\mathrm{a}\mathrm{x}}$$

2. Voltage regulation requirement: D-STATCOM operation must ensure voltage at each bus remains within statutory limits (e.g., ± 5% of nominal).

3. Thermal loading constraints: Apparent power flow through each line must not exceed its thermal rating:

∣Sij∣ ≤ Sijmax.

4. Feasibility of AC power flow: D-STATCOM set points must yield a convergent AC load flow solution under all considered uncertainty scenarios.

These criteria are enforced during each NSGA-II iteration via AC load flow feasibility checks implemented in MATPOWER. Candidate solutions violating any of the above constraints are penalized or discarded, ensuring that all reported Pareto-optimal solutions satisfy the operational and engineering requirements for D-STATCOM installation.

e) Thermal Limits

$$\left|{\mathrm{I}}_{\mathrm{l}}\right| \le {\mathrm{I}}_{\mathrm{m}\mathrm{a}\mathrm{x},\mathrm{l}}$$

The above constraints represent standard AC power flow and operational limits commonly adopted in distribution system optimization. Power balance equations, voltage magnitude limits, DER and D-STATCOM capacity bounds, and thermal line constraints are enforced during each optimization iteration through the AC load flow solution implemented in MATPOWER. Candidate solutions violating any constraint are discarded or penalized within the NSGA-II fitness evaluation, thereby ensuring that all reported Pareto-optimal solutions satisfy the formal mathematical constraints of the model.

#### Sub-objectives

- Technical

Minimizes active power losses, voltage deviations, and line loading^6,12^.

15 $${f}_{tech}= \sum {P}_{loss} + \sum {\left(1-{V}_{i}\right)}^{2}+ \sum \frac{{S}_{ij}}{{S}_{ij}^{max}}$$

b) Economic

Minimizes the total cost of investment and operation while accounting for the financial savings from reduced losses^4,6^.

16 $${f}_{eco}=CAPEX+OPEX- {S}_{loss,saving}$$

c) Environmental (including LCA)

Minimizes the total life-cycle CO₂ emissions, as defined by the LCA model^23^.

17 $${f}_{env}={CO}_{2,total}= \sum {E}_{i} X E{F}_{i}$$

d) Social

Maximizes job creation (J) and minimizes grid dependency (GD) to enhance local socio-economic value^21,22^.

18 $$ss= {\alpha}_{1} J+ {\alpha}_{2} ( 1-GD)$$

The social score defined in Eq. (18) combines job creation and grid dependency indicators. Job creation is estimated using the employment factor method reported in the literature^22^, expressed in full-time equivalent positions per installed MW of renewable capacity. Grid dependency is defined as the ratio of imported grid energy to total supplied energy. The weighting coefficients reflect a higher priority assigned to local economic development within the Egyptian energy context, consistent with national energy policy objectives. Grid dependency (GD) is quantified as the ratio of imported energy to total demand, with coefficients α₁ = 0.7 and α₂ = 0.3 determined through expert weighting for the Egyptian context. The adopted weighting factors reflect expert judgment aligned with Egyptian socio-economic priorities and are therefore context-specific. When applying the proposed framework to other emerging economies, these coefficients can be readily adjusted through stakeholder consultation or sensitivity analysis to reflect local policy objectives, labor market structures, and energy security considerations. This flexibility preserves the general applicability of the framework while maintaining transparency in social impact assessment. A complete listing of all parameters used in the probabilistic models, economic calculations, life-cycle assessment, and optimization algorithm is provided in the Supplementary File.

## Optimization framework algorithm design and pareto analysis

The Non-Dominated Sorting Genetic Algorithm II (NSGA-II) is employed to solve this complex MOO problem. NSGA-II is a proven metaheuristic algorithm effective for power system optimization, known for its ability to efficiently converge upon a high-quality, diverse set of Pareto-optimal solutions [7, 12 and 13]. The algorithm utilizes non-dominated sorting and a crowding distance mechanism to navigate the trade-offs between the technical, economic, environmental, and social objectives.

### Proposed optimization framework flowchart

**Fig. 2:**
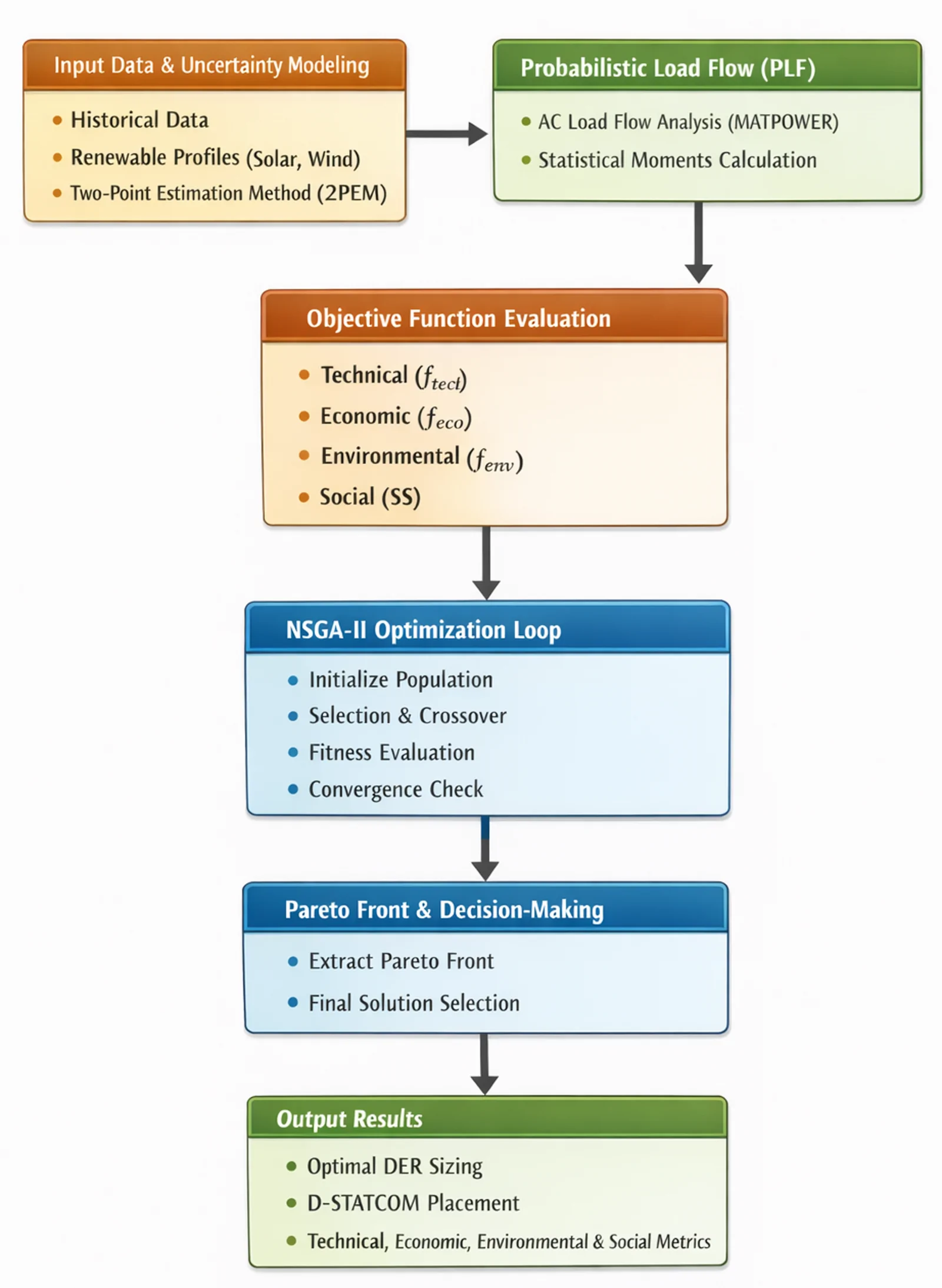
Proposed probabilistic multi-objective optimization framework for DER sizing and D-STATCOM planning under uncertainty.

The proposed probabilistic multi-objective optimization framework is illustrated in Fig. 2, which outlines the integrated workflow from uncertainty modeling to final decision-making. The flowchart consists of the following key stages:

#### Input data & uncertainty modeling

I. Load historical data, renewable generation profiles (solar irradiance, wind speed), and system parameters.
II. Apply the Two-Point Estimation Method (2PEM) to model uncertainties in load, PV, and wind generation.

#### Probabilistic load flow (PLF)

I. For each 2PEM scenario, perform AC load flow using MATPOWER.
II. Compute statistical moments of bus voltages, line flows, and losses.

#### Objective function evaluation

I. Evaluate the four objective functions:
II. Technical (f_tech_): losses, voltage deviation, loading.
III. Economic (f_eco_): CAPEX, OPEX, loss savings.
IV. Environmental (f_env_): life-cycle CO₂ emissions (LCA).
V. Social (SS): job creation, grid dependency.
VI. Check constraints: voltage limits, thermal limits, DER/D-STATCOM capacities.

#### NSGA-II optimization loop

I. Initialize population within feasible bounds.
II. Evaluate fitness for all candidates.
III. Non-dominated sorting to rank solutions.
IV. Crowding distance calculation to maintain diversity.
V. Selection, crossover, mutation to generate new population.
VI. Repeat until convergence criteria met.

#### Pareto front & decision-making

I. Extract non-dominated solutions (Pareto front).
II. Apply decision-making method to select the final optimal compromise solution.

#### Output

Optimal DER sizing, D-STATCOM placement and sizing, and performance metrics across technical, economic, environmental, and social dimensions.

### Performance metrics

The performance of the optimized microgrid configurations is evaluated using standardized metrics that align with the four objective dimensions. Performance indicators (Eqs. 19–22) include LCOE, SPP, CO₂ reduction and grid dependency.

#### Levelized cost of energy (LCOE)^4^

19 $$LCOE=\frac{\sum_{t=1}^{N}({I}_{t} + {O}_{t} + {F}_{t})}{\sum_{t=1}^{N}({E}_{t})}$$

#### Simple payback period (SPP)^4^

20 $$SSP=\frac{\mathrm{I}\mathrm{n}\mathrm{v}\mathrm{e}\mathrm{s}\mathrm{t}\mathrm{m}\mathrm{e}\mathrm{n}\mathrm{t}}{\mathrm{A}\mathrm{n}\mathrm{n}\mathrm{u}\mathrm{a}\mathrm{l} \mathrm{N}\mathrm{e}\mathrm{t} \mathrm{S}\mathrm{a}\mathrm{v}\mathrm{i}\mathrm{n}\mathrm{g}\mathrm{s}}$$

#### CO₂ reduction efficiency^23^

21 $${\eta}_{CO2}= \frac{{E}_{base}-{E}_{Opt}}{{E}_{base}} x100$$

#### Grid dependency ratio^21,22,29^

22 $$GD= \frac{{E}_{import}}{{E}_{total}}$$

## Results and discussion

This section presents a comprehensive analysis of the probabilistic optimization outcomes for a grid-connected microgrid, accounting for uncertainties in wind, PV, and load demand. Although a benchmark IEEE-33 bus system is employed, Egyptian distribution network conditions are represented through a worst-case loading scenario corresponding to 115% of base demand, as reported for stressed feeders in Egypt^5^. Moreover, real solar irradiance and wind speed data from the Red Sea region are used to model renewable generation uncertainty. This approach enables realistic assessment of DER-rich Egyptian grids without relying on confidential utility feeder data. High-resolution meteorological data from the Red Sea coast inform the renewable generation models. System performance is evaluated over a 20-year horizon across four key dimensions: technical, economic, environmental, and social. The analysis systematically compares the base grid (no DERs), an optimally sized DER configuration, and enhancements via IEEE 1547–2018-compliant inverter capabilities, culminating in a detailed comparison of five reactive power compensation strategies.

### Optimal DER configuration and pareto front analysis

**Fig. 3:**
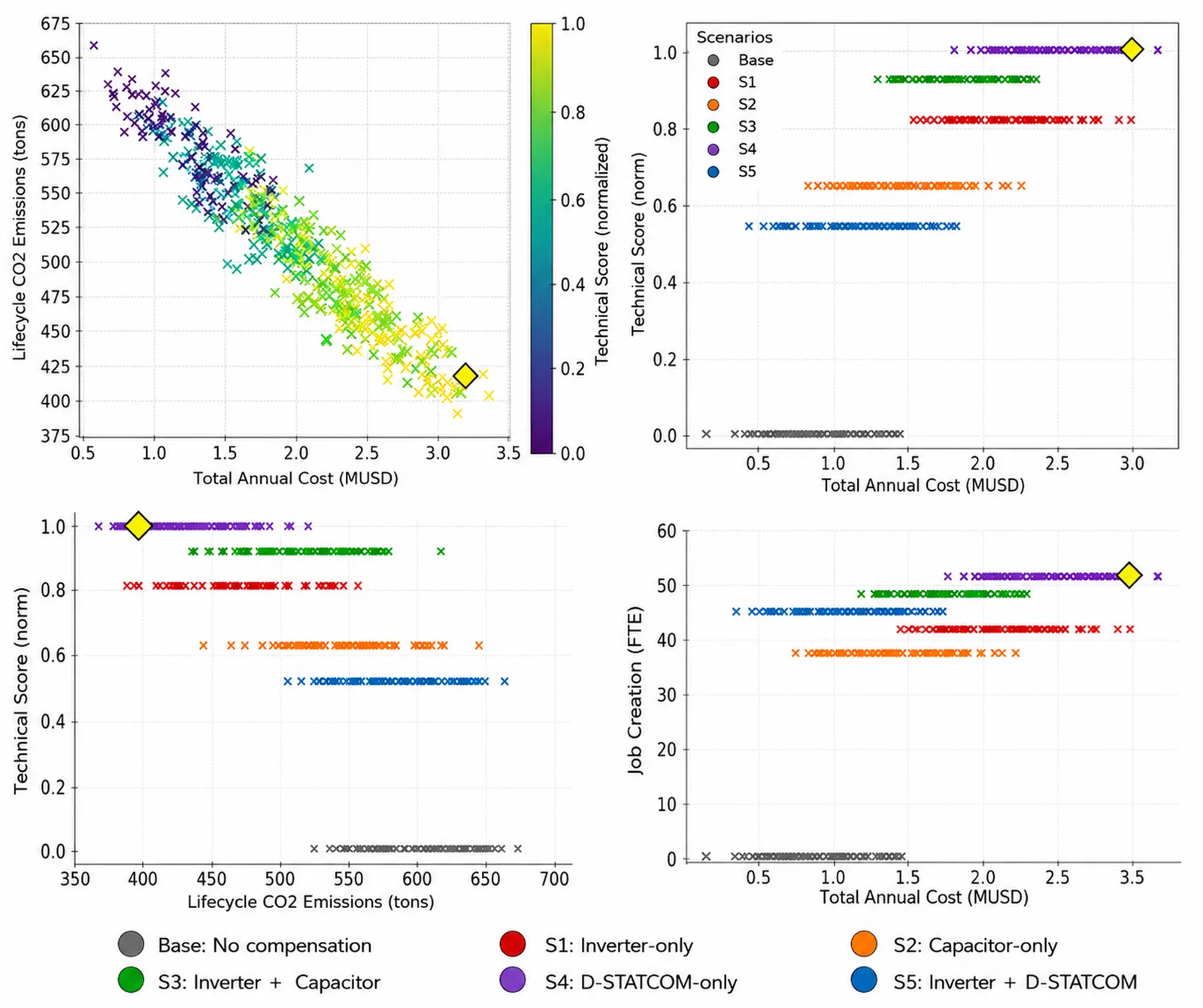
Multi-objective trade-offs and the selected optimal solution for reactive power planning

The base IEEE 33-bus system exhibits the benchmark performance metrics was discussed in Section "Base system configuration and benchmark metrics", minimum voltage of 0.927 p.u. at bus 18, total active losses of 185.6 kW, and a maximum line loading of 148.2%. Under the 115% stressed loading scenario representing Egyptian distribution conditions, these values degrade further, highlighting the need for intervention. The optimal DER portfolio (4 MW PV, 2.9 MW Wind, 1 MW Diesel) improves the minimum voltage to 0.952 p.u., reduces losses by 56.4% to 81.2 kW, and cuts grid imports by 74.7%. However, voltage stability remains inadequate, necessitating dedicated reactive power compensation. Figure 3 shows Multi-objective trade-off analysis showing the relationship between total annual cost, life-cycle CO₂ emissions, technical performance, and job creation. Different colors and marker styles represent the evaluated scenarios (Base, S1–S5), as defined in the legend Base: No compensation S1: Inverter-only S2: Capacitor-only S3: Inverter + Capacitor S4: D-STATCOM-only S5: Inverter + D-STATCOM. The color scale indicates the normalized technical score, where higher values denote improved voltage profile and loss reduction. The highlighted marker denotes the selected optimal compromise solution.

It is important to note that the convergence behavior of NSGA-II in complex microgrid planning problems may be influenced by several interacting factors. In the present study, the high dimensionality and non-convex nature of the formulated four-dimensional optimization problem combining probabilistic technical constraints with economic, environmental, and social objectives significantly increases the complexity of the search space and may lead to convergence toward locally optimal Pareto fronts. Although NSGA-II incorporates non-dominated sorting and crowding distance mechanisms to preserve solution diversity, its performance remains sensitive to algorithmic parameters such as population size, crossover and mutation rates, and termination criteria. To mitigate premature convergence, parameter values were selected based on established recommendations in the literature, and multiple independent runs were performed to ensure the stability and repeatability of the obtained Pareto fronts. Accordingly, the reported solutions should be interpreted as robust compromise solutions rather than guaranteed global optima.

### Technical performance of reactive power compensation

**Fig. 4:**
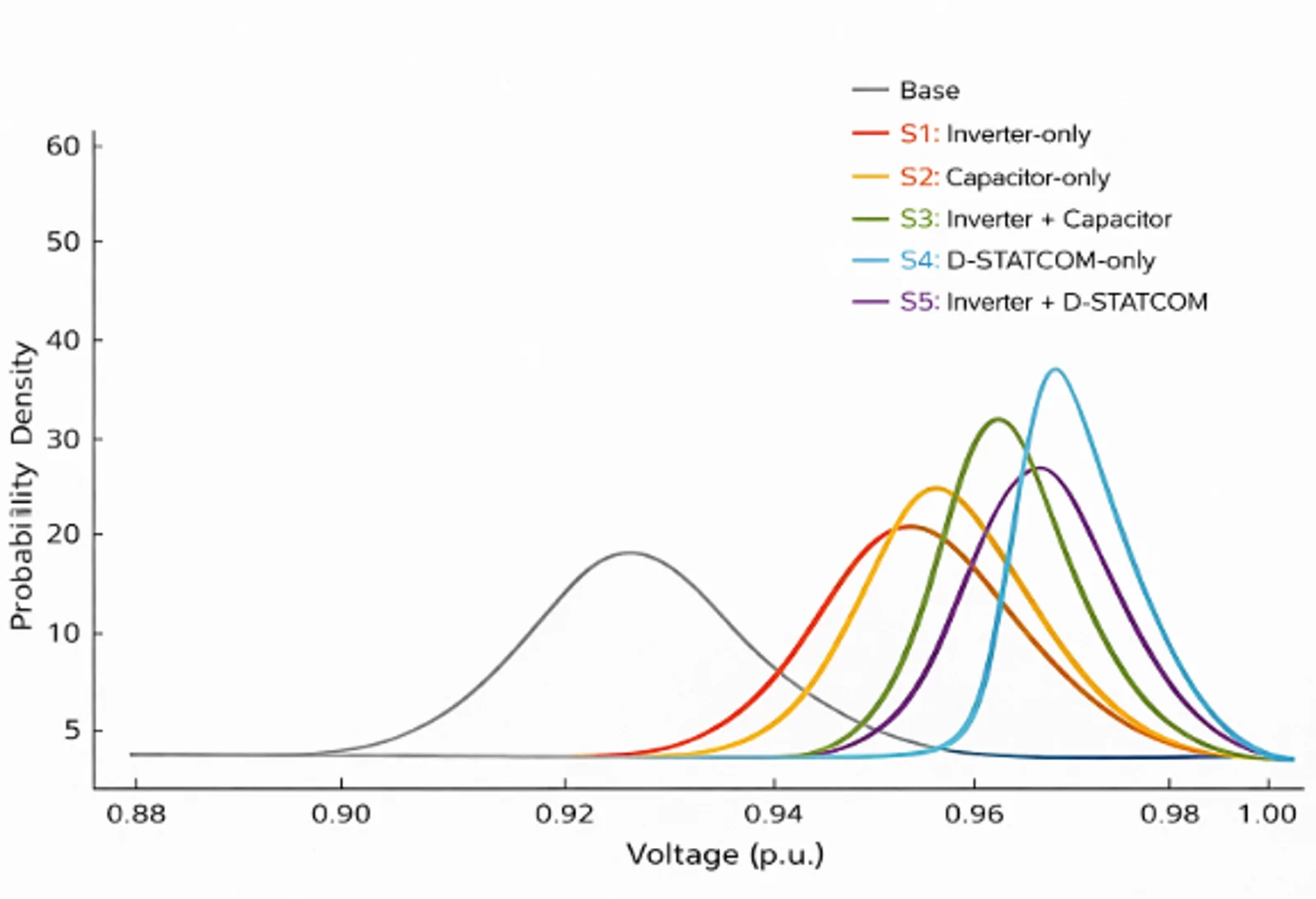
Probability density functions (PDFs) of bus voltages comparing the base case and the proposed reactive power planning scenarios under probabilistic optimization.

**Fig. 5:**
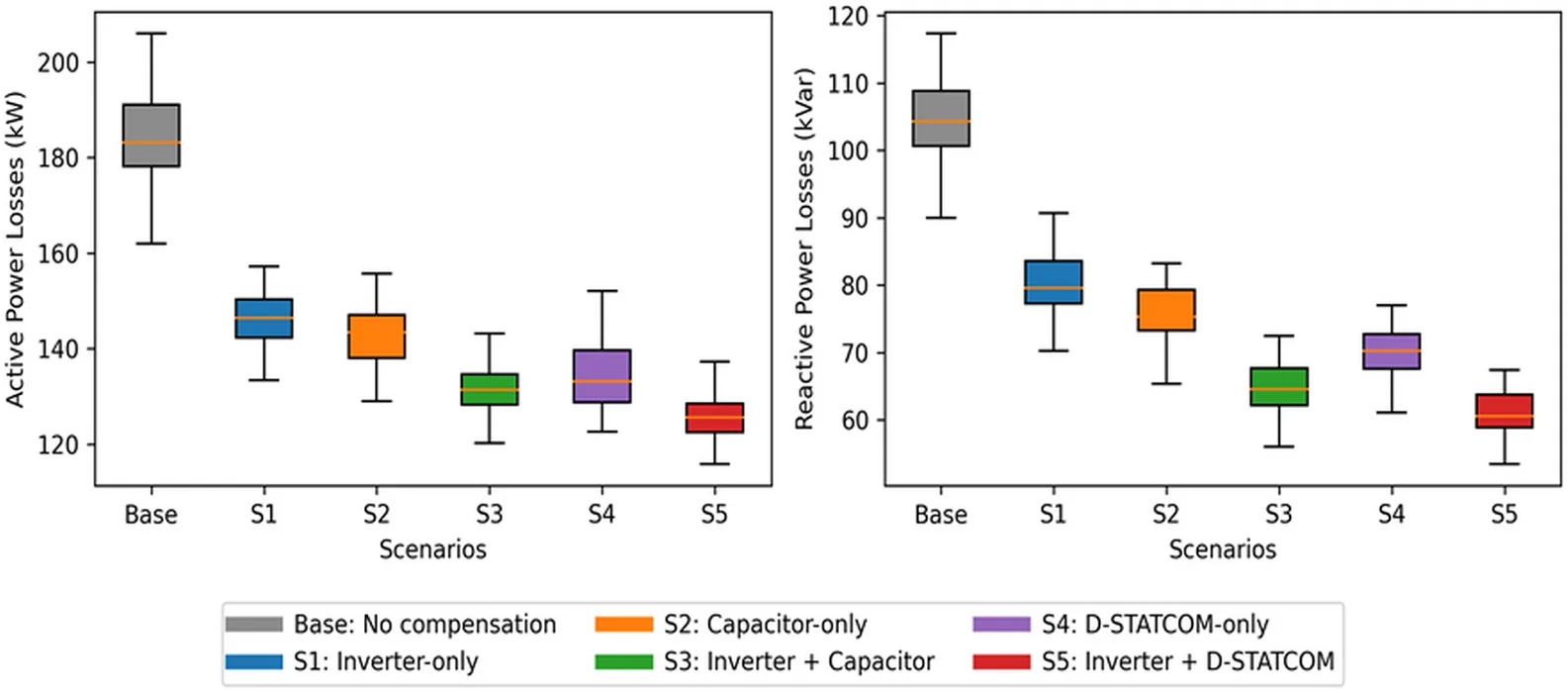
Statistical distribution of active and reactive power losses across reactive power planning scenarios

The base case represents conventional grid operation without any distributed energy resources (DERs) or dedicated reactive power compensation devices. In base case scenario, voltage stability is solely dependent on the grid supply, resulting in frequent under-voltage events and high variability under stochastic loading conditions. In contrast, the proposed reactive power planning framework integrates probabilistically optimized DER sizing with coordinated reactive compensation strategies, progressively shifting and narrowing the voltage distributions as shown in Fig. 4. Each scenario (S1–S5) corresponds to a specific planning strategy ranging from inverter only support to combination of inverter + D-STATCOM configurations optimized under the same uncertainty aware multi-objective framework. The progressive rightward shift and reduced variance of the voltage PDFs demonstrate the effectiveness of the proposed approach in enhancing voltage stability and mitigating violations under renewable and load uncertainty**.** The voltage probability distributions in Fig. 4 illustrate the progressive improvement achieved by the proposed reactive power planning framework relative to the base case. The base case, representing conventional grid operation without DERs or reactive sources, exhibits a left-skewed PDF with significant mass below 0.95 p.u., indicating frequent under-voltage conditions. In contrast, each optimized scenario (S1–S5) shifts the distribution toward higher voltages and reduces its variance, reflecting enhanced voltage stability. The combination of Inverter + D-STATCOM scenario (S5) yields the narrowest, most right-shifted PDF, confirming that the proposed probabilistic co-optimization of DER sizing and reactive compensation effectively mitigates voltage violations even under stochastic renewable and load variations**.** The proposed scenarios shows in Fig. 4 progressively shift the distribution rightward and narrow its spread, with the proposed combination of Inverter + D-STATCOM configuration (S5) achieving the best distribution around the nominal voltage, demonstrating superior voltage regulation under uncertainty. This is complemented by the loss analysis in Fig. 5, where S5 achieves both the lowest median and smallest variance in active and reactive power losses, Confirming its superior efficiency and operational consistency over alternative scenarios. Although reactive compensation devices are deployed to support voltage regulation and reduce reactive power flows, reactive power losses cannot be entirely eliminated in practical distribution systems. This residual loss is due to the inherent inductive reactance of feeders, non-zero current magnitude required to deliver active power, and the load-dependent reactive demand that varies with stochastic generation and consumption. As demonstrated in Fig. 5, while compensation strategies especially the combination of Inverter + D-STATCOM configuration (S5) significantly reduce both the median and variance of reactive losses compared to the base case, they do not drive reactive losses to zero. This outcome is consistent with real-world operation, where compensation devices are optimized to enhance voltage stability, minimize loss magnitude, and improve system efficiency under uncertainty, rather than to achieve complete cancellation of reactive power flow across all operating points.

Table 2 shows the base case demonstrates the poorest performance, characterized by severe under voltage, feeder overload, and high losses. Activating the inherent reactive capability of the inverters (S1) improved the minimum voltage by 3.3% and reduced line loading by 48.1%. However, this reallocation of inverter capacity comes at the expense of active power curtailment. Scenario 2 (capacitor banks) provided a static, cost-effective boost, achieving a 3.7% voltage rise. The hybrid configurations (S3 and S5) proved superior. Scenario 3 delivered a 4.8% voltage improvement and significant loss reduction, while Scenario 5 emerged as the most technically robust, achieving the best voltage profile (0.975 p.u.), the lowest line loading (68.4%), and the most substantial reduction in both active (31.7%) and reactive (41.7%) losses.

**Table 2:** Technical Performance of Compensation Scenarios.

Scenario	Strategy	Sizing	Worst voltage (p.u.)	Worst line loading (%)	Active losses (kW)	Reactive losses (kVar)
Base Case	None	Grid supply only	0.927	148.2	185.6	105.2
S1	Inverter Q Support	31.2% of 7.6 MVA inverter capacity	0.958	76.9	148.3	79.2
S2	Capacitor Banks	4.0 MVAr at Bus 12, 4.0 MVAr at Bus 30	0.961	75.3	142.7	74.5
S3	Inverter + Capacitor	5.0 MVAr capacitors at Bus 30 with inverter support	0.972	69.7	129.5	64.1
S4	D-STATCOM Only	4.5 MVAr at Bus 12, 2.25 MVAr at Bus 30	0.968	71.8	134.2	68.9
S5	Inverter + D-STATCOM	2.5 MVAr D-STATCOM at Bus 30 with inverter support	0.975	68.4	126.8	61.3

### Economic viability and financial analysis

A comprehensive economic analysis was conducted to evaluate the financial merits of each compensation strategy, with results detailed in Table 3. The analysis considers total capital expenditure (CAPEX), operational and maintenance costs (OPEX), Simple Payback Period (SPP), Internal Rate of Return (IRR), and the Levelized Cost of Energy (LCOE).

**Table 3:** Economic Analysis of Investment Scenarios.

Scenario	Total Capital ($M)	Total O&M ($K/yr)	Payback (years)	IRR (%)	LCOE ($/kWh)
Base case	0.0	65.4	-	-	0.142
S1 (inverter only)	7.95	121.0	8.5	11.2	0.095
S2 (capacitor only)	8.20	132.0	7.6	13.2	0.086
S3 (capacitor + inverter)	8.70	136.0	8.8	10.8	0.093
S4 (DSTATCOM only)	8.58	154.0	8.1	11.9	0.089
S5 (DSTATCOM + Inverter)	8.83	143.0	9.2	10.1	0.091

The economic analysis in Table 3 also reveals distinct investment trade-offs. The capacitor-only strategy (S2) offers the most favorable standalone financials, with the lowest capital cost, shortest payback (7.6 years), and highest IRR (13.2%). In contrast, hybrid configurations (S3, S5) incur a 6–8% capital premium, reflected in longer payback periods. Specifically, the optimal S5 scenario has the longest payback (9.2 years) and lowest IRR (10.1%), a cost justified by its superior technical resilience and operational savings, as established in Sect. 5.2. Critically, all DER-integrated scenarios achieve a significantly lower LCOE than the base case, confirming the long-term economic advantage of renewable integration.

### Sustainability and socio-economic outcomes

**Fig. 6:**
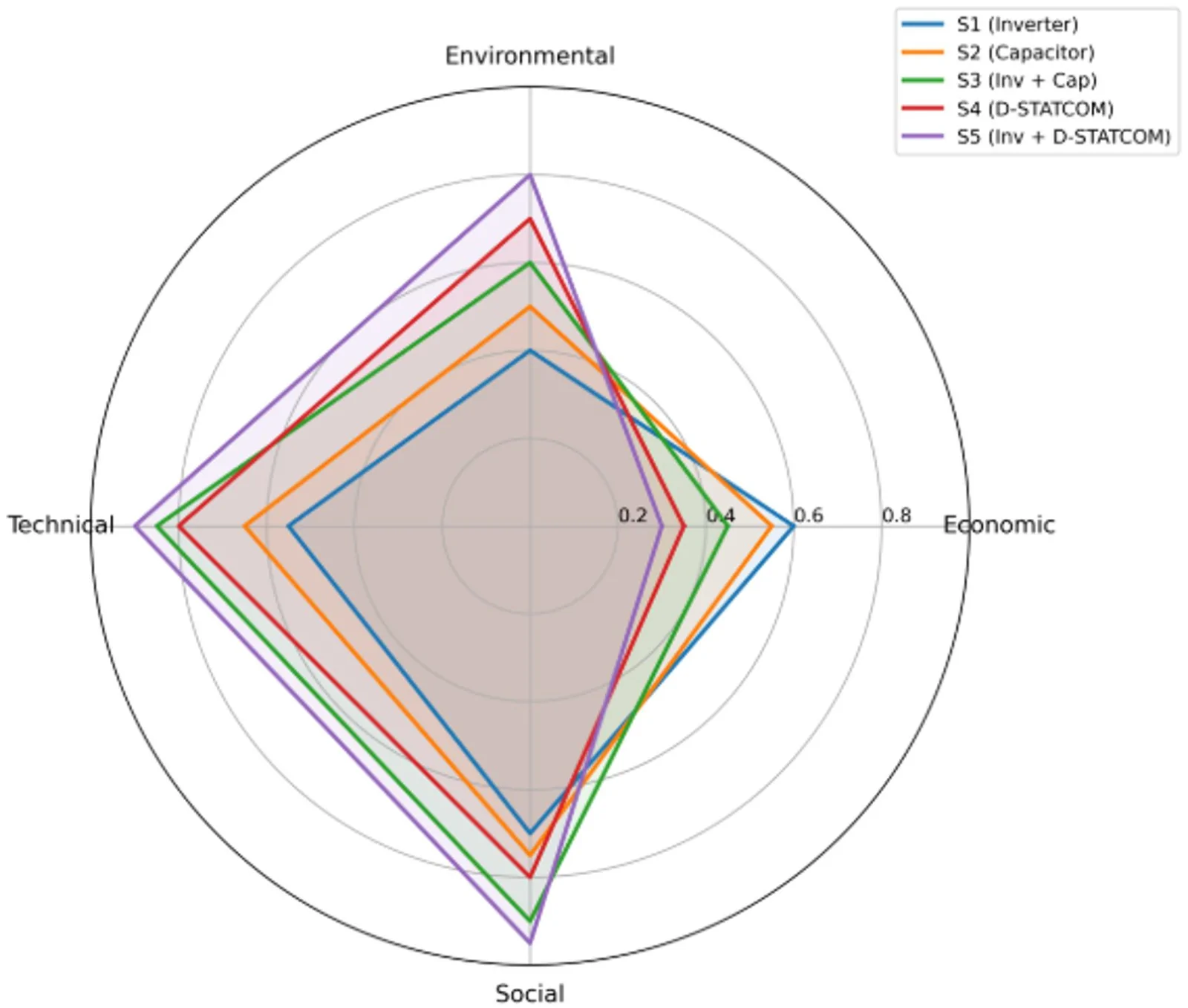
Normalized radar chart comparing the economic, environmental, technical, and social performance of all evaluated scenarios

The broader impacts on sustainability and social criteria are presented in Table 4, capturing CO₂ reduction, grid dependency, job creation, and ancillary financial benefits from loss savings and carbon reduction. Figure 6 Integrated sustainability performance comparison of all evaluated scenarios using a normalized radar chart. The four axes represent economic performance (investment and operating cost indicators), environmental performance (life-cycle CO₂ reduction), technical performance (voltage profile improvement and loss reduction), and social performance (job creation and grid dependency reduction). Scenarios forming larger and more balanced polygons demonstrate stronger integrated performance, while narrower shapes indicate trade-offs favoring specific objectives .All indicators are normalized on a 0–1 scale to allow direct comparison across scenarios, where a larger enclosed area indicates superior overall performance. Scenario labels and colors are defined in the legend. While S1 (Inverter Only) excels in environmental performance, it shows weaknesses in economic and technical dimensions. In contrast, the optimal S5 configuration forms the largest, most balanced polygon, demonstrating excellence in technical performance and social benefits (job creation), with competitive scores in economic and environmental criteria. This integrated view substantiates the selection of S5 as the most balanced solution, effectively reconciling the often-conflicting goals of cost, emissions, reliability, and social value.

**Table 4:** Comprehensive Sustainability Metrics Analysis.

Scenario	CO₂ Reduction (%)	Grid dependency (%)	Job creation (FTE)	Carbon savings ($/yr)	Loss savings ($/yr)
Base Case	0	100	0	0	0
S1	65.4	35	45	9,785	39,300
S2	63.8	32	38	9,485	45,200
S3	62.6	25	48	9,300	59,100
S4	61.2	28	42	9,115	54,100
S5	59.8	22	52	8,865	61,900

Scenario 1 (Inverter Only) achieves the highest CO₂ reduction (65.4%) but results in higher grid dependency (35%). The hybrid scenarios excel in enhancing energy independence and stimulating local economies. Scenario 5, in particular, minimizes grid dependency to 22% and generates the highest number of full-time equivalent jobs (52 FTE), while also delivering the greatest annual savings from reduced power losses ($61,900). This positions it as the optimal strategy for maximizing socio-economic resilience, despite a marginally lower CO₂ reduction percentage.

Compared with recent state-of-the-art approaches reported in the literature, which typically address reactive power planning using deterministic optimization or focus on isolated technical or economic objectives, the proposed framework demonstrates clear advantages. By integrating probabilistic uncertainty modeling with multi-objective optimization, the presented results show enhanced robustness against renewable variability and load fluctuations. Furthermore, the inclusion of life-cycle environmental impact and social indicators enables a more comprehensive assessment than conventional techno-economic methods. The comparative scenario analysis confirms that hybrid inverter–D-STATCOM solutions consistently outperform single-device or static compensation strategies across multiple performance dimensions under identical operating conditions. From a policymaker’s perspective in Egypt, grid modernization requires prioritizing not only emissions reduction but also voltage compliance, operational resilience, and energy security in heavily loaded distribution networks. While the inverter-only strategy (S1) maximizes CO₂ reduction, the combination of inverter + D-STATCOM strategy (S5) offers a more balanced pathway by simultaneously enhancing voltage stability, reducing grid dependency, and maximizing socio-economic benefits. The proposed Pareto-based framework explicitly supports such policy driven decision making by allowing stakeholders to transparently negotiate trade-offs among environmental, technical, economic, and social objectives, rather than enforcing a single-objective optimum.

### Sensitivity analysis

To assess the robustness of scenario rankings under economic uncertainty, a comprehensive sensitivity analysis was performed considering technology cost variations (± 30%), energy price fluctuations (± 20%), carbon credit valuation ($25–$75/ton CO₂), discount rate variation (5–15%), and capital scenarios (0 – 40%). These parameters were selected based on their relevance to Egyptian market conditions and emerging economy benchmarks reported in recent literature^4,5,18,30^.The results confirm that the hybrid inverter + D-STATCOM strategy (S5) remains the preferred solution across most plausible economic futures when technical, social, and environmental objectives are jointly considered. This finding aligns with recent studies demonstrating the robustness of hybrid compensation schemes under uncertainty^30,32^. The capacitor-only strategy (S2), while financially attractive under baseline assumptions, is highly sensitive to energy price reductions and loses its advantage when carbon pricing or social benefits are included consistent with observations that static compensation devices offer limited adaptability under varying market conditions^31,33^.These findings reinforce the robustness of S5 as a balanced, long-term investment for grid modernization under policy and market uncertainty. Detailed sensitivity analysis results, including complete scenario tables and tornado charts, are provided in the Supplementary File.

## Model validation and benchmark comparison

To ensure the reliability and credibility of the proposed probabilistic multi-objective optimization framework, the obtained results were validated through comparison with benchmark studies reported in the literature using the widely adopted IEEE-33 bus distribution system. First, the base case operating conditions obtained in this study are consistent with the standard benchmark values reported in previous literature for the IEEE-33 bus radial distribution system. The obtained base case results show a minimum bus voltage of **0**.927 p.u., total active power losses of 185.6 kW, and significant feeder overloading exceeding 148%, which closely match the benchmark values reported in previous studies using the same test system^6^. This consistency confirms the correctness of the implemented AC power flow model and the adopted system configuration.

Second, the effectiveness of the proposed optimization framework was validated by comparing the obtained improvements with results reported in recent studies employing advanced metaheuristic optimization algorithms for power system applications. Recent research has demonstrated that hybrid and swarm-based optimization algorithms can effectively solve complex nonlinear optimization problems in power transmission and distribution networks^30,31^. In particular, hybrid optimization frameworks combining different metaheuristic strategies have shown improved convergence characteristics and enhanced global search capabilities compared to conventional optimization approaches. In addition, comprehensive reviews of modern metaheuristic algorithms highlight their suitability for addressing large-scale multi-objective optimization problems in energy systems due to their ability to balance exploration and exploitation in complex search spaces^32^. These findings support the selection of evolutionary optimization techniques such as NSGA-II for solving the formulated multi-objective planning problem. The optimization results obtained in this study are consistent with these observations. The proposed probabilistic framework significantly improves system performance compared with the base grid configuration. In particular, the optimal Inverter + D-STATCOM hybrid configuration (S5**)** improves the minimum bus voltage to 0.975 p.u**.**, reduces active power losses by approximately 31.7%, and decreases the maximum feeder loading to 68.4%. These improvements confirm the effectiveness of coordinated reactive power planning in DER-integrated distribution networks.

Furthermore, recent studies have shown that enhanced swarm-based optimization algorithms incorporating chaotic search mechanisms can improve exploration capability and optimization stability in complex engineering problems^33^. The consistent performance improvements observed in the present study further confirm the robustness of the proposed multi-objective optimization framework. Overall, the agreement between the obtained benchmark results and previously reported studies confirms the validity of the proposed modeling framework and demonstrates the effectiveness of the proposed probabilistic multi-objective optimization approach for sustainable microgrid planning.

## Algorithm robustness analysis

To further evaluate the robustness of the proposed optimization framework, multiple independent runs of the NSGA-II algorithm were conducted under identical system conditions. Due to the stochastic nature of evolutionary optimization algorithms, slight variations in the obtained Pareto solutions may occur depending on the initial population and genetic operations. However, the obtained Pareto fronts across multiple runs showed consistent convergence behavior and similar distributions of optimal solutions. In particular, the hybrid Inverter + D-STATCOM configuration (S5**)** consistently appeared among the dominant Pareto solutions across independent optimization runs, confirming the stability of the obtained optimal strategy. These observations are consistent with findings reported in the literature, where metaheuristic optimization algorithms have demonstrated strong robustness and reliable convergence behavior when applied to complex engineering optimization problems in power systems and distribution networks^30–33^. Therefore, the results confirm that the proposed probabilistic multi-objective optimization framework provides stable and reliable solutions for DER-integrated microgrid planning problems.

## Conclusion and future research

This study presented a probabilistic multi-objective optimization framework for reactive power planning in DER-integrated distribution networks, jointly considering technical, economic, environmental, and social objectives under uncertainty. The results highlight a fundamental trade-off between maximizing environmental benefits and ensuring techno-economic resilience. While the inverter-only strategy (S1) achieves the highest CO₂ reduction, it compromises active power utilization and long-term economic efficiency due to inverter curtailment. The capacitor-only solution (S2) offers the most attractive financial metrics but lacks dynamic response capability. Hybrid strategies provide more balanced system performance. In particular, the Inverter + D-STATCOM configuration (S5) emerges as the most comprehensive solution for technically demanding networks, delivering superior voltage stability, the lowest grid dependency, the highest job creation, and the largest operational savings from loss reduction despite a slightly higher investment cost. Consequently, inverter-only solutions may be suitable for cost-sensitive deployments, whereas hybrid configurations especially Inverter + D-STATCOM are recommended for weak or highly loaded grids where reliability, energy independence, and socio-economic benefits are critical. These findings indicate that coordinated inverter and D-STATCOM deployment is a promising strategy for renewable-rich distribution networks, particularly in emerging economies.

Future research should extend the proposed framework to real large-scale distribution feeders, integrate energy storage systems and demand response, and investigate adaptive or real-time control strategies under dynamic operating conditions. Further developments may incorporate resilience metrics, extreme-event analysis, transient stability constraints within the optimization loop, and dynamic models of inverter-based resources to evaluate system performance under grid disturbances. Statistical validation using non-parametric methods, such as the Wilcoxon signed-rank test, could further quantify the confidence of performance differences using extensive time-series data. Practical implementation on real Egyptian feeders will require addressing challenges related to data availability, computational complexity, and integration with existing utility planning practices; these challenges can be mitigated through feeder zoning, network reduction techniques, parallel computing, and staged pilot deployments. In addition, future sustainability assessments could expand life-cycle indicators to include water consumption, land-use footprint, and material criticality. Finally, future implementations should investigate hierarchical control architectures combining primary local droop control with secondary centralized optimization operating every 5–15 min, supported by controller hardware-in-the-loop validation prior to field deployment.

## Supplementary Information

Below is the link to the electronic supplementary material.

Supplementary Material 1

Supplementary Material 2

## Data Availability

The data underlying this article are available in the article.

## Parameters (Fixed Inputs, Coefficients, Limits)

P: Active power (kW)
Q: Reactive power (kVar)
Sijmax​: Thermal limit of line ij (kVA)
Ilmax: Maximum allowable current in line l (A)
Vmin ,Vmax: Minimum and maximum voltage limits (p.u.)
E[Y]: Expected value of random variable Y (—)
var[Y]: Variance of random variable Y (—)
ωi: Weighting factor in Two-Point Estimation Method (2PEM) (—)
G: Solar irradiance (W/m^2^)
GSTC: Standard solar irradiance at STC (1000 W/m^2^)
Ta: Ambient temperature (°C)
TS,TC: Temperature at standard test conditions (25 °C)
α: Temperature coefficient of the PV module (1/°C)
PSTC: Rated PV power at STC (kW)
NOCT: Nominal Operating Cell Temperature (°C)
v: Wind speed (m/s)
Vci ,Vr ,Vco: Cut-in, rated, and cut-out wind speeds (m/s)
Pr: Rated wind turbine power (kW)
ρ(v): Weibull probability density function for wind speed (—)
k, c: Weibull shape and scale parameters (—)
Mi: Mass or rated capacity of component i (kg or kW)
EFM ,i: Emission factor for manufacturing stage (ton CO₂/unit)
EFT, j: Emission factor for transport stage (ton CO₂/unit)
EFINS,EFOPR,EFD: Emission factors for installation, operation, decommissioning (ton CO₂/unit)
Dj: Transport distance for component j (km)
Wj: Weight of component j (kg)
RREC: Recycled fraction / avoided emissions (%)
w1,w2,w3,w4: Weighting coefficients for objectives (—)
α1,α2: Social weighting factors for job and dependency (—)
P0: Initial population in NSGA-II (—)
fmax, fmin: Maximum and minimum objective values (—)
LCA: Life cycle assessment (—)

## Variables

Vi: Voltage magnitude at bus i (p.u. )
θi: Voltage angle at bus i (Radians)
Sij: Apparent power flow in line ij (kVA)
Il: Current magnitude in line l (A)
Pg, Qg: Generated active and reactive power (kW, kVar)
Ploss, Qloss: Total active and reactive losses (kW, kVar)
TC: PV cell temperature (°C)
PWT: Wind turbine power output (kW)
E[Vi]: Expected voltage at bus i (p.u. )
σVi: Standard deviation of voltage at bus i (p.u. )
ELCA: Total life-cycle emissions (ton CO₂-eq)
EMFG: Emissions from manufacturing stage (ton CO₂-eq)
ETRN: Emissions from transportation stage (ton CO₂-eq)
EINS: Emissions from installation and construction (ton CO₂-eq)
EOPR: Emissions from operation and maintenance (ton CO₂-eq)
EDEC: Emissions from decommissioning/recycling (ton CO₂-eq)
Egen,t: Generated energy at time t (kWh)
EFLCA: Emission intensity per kWh (ton CO₂/kWh)
F(x): Overall multi-objective optimization function (—)
Ftech: Technical objective (losses, voltage deviation, loading) (—)
Feco: Economic objective (CAPEX + OPEXSavings) (—)
Fenv: Environmental objective (total CO₂ emissions) (—)
SS: Social score (—)
PDER: Active power of distributed energy resource (kW)
QDER: Reactive power of distributed energy resource (kVar)
CAPEX: Capital cost of DERs and compensation devices ($)
OPEX: Operational and maintenance cost ($/year)
Sloss, saving: Annual savings from reduced power losses ($/year)
CO2, totalCO2: Total CO₂ emissions (ton CO₂)
J: Job creation (Full-Time Equivalent positions) (FTE)
GD: Grid dependency ratio (%)
xa, xb: Candidate solutions (—)
fi(x): Objective function value for solution x (—)
CDi: Crowding distance of solution i (—)
LCOE: Levelized Cost of Energy ($/kWh)
SPP: Simple Payback Period (Years)
ηCO2: CO₂ reduction efficiency (%)
Ebase, EOpt: Base and optimized CO₂ emissions (ton CO₂)
Eimport: Imported grid energy (kWh)
Etotal: Total supplied energy (kWh)
ΔVi: Voltage deviation at bus i (p.u. )
MOO: Multi Objective Optimization (-)

## Indices

N: Number of buses or time steps
M: Number of objectives in NSGA-II
i, j: Bus and line indices
t: Time index
N: Number of random variables (2PEM)
K: Scenario index
M,T,INS,OPR,DEC: Life-cycle stages: Manufacturing, Transport, Installation, Operation, Decommissioning
