# Supplementary material for: A probabilistic multi objective decision framework for reactive power planning in der integrated microgrids enhancing sustainability and resilience in the egyptian grid
Source: Sci Rep. 2026 Aug 20;16:26167. doi: 10.1038/s41598-026-66944-w (PMC13494049; doi:10.1038/s41598-026-66944-w)
Supplement: Supplementary file 1 [file 41598_2026_66944_MOESM1_ESM.docx]

**Case Study Parameters and Input Data**

The probabilistic simulations and economic models rely on a set of well-defined parameters to ensure the results are realistic and applicable to the Egyptian context. These parameters, summarized in Table 1 and 2 , are derived from manufacturer specifications, high-resolution Egyptian meteorological data, international lifecycle assessment (LCA) databases, and national grid standards. The use of these standardized and cited values ensures the reproducibility and validity of the optimization framework.

**Table 1: Key Parameters for Probabilistic Simulation and Economic Modeling**

| **Parameter** | **Symbol** | **Value / Range** | **Unit** | **Source / Justification** |
| --- | --- | --- | --- | --- |
| **A. Probabilistic & Renewable Models** | | | | |
| **Weibull shape parameter (Wind)** | k | 2.1 | --- | Fitted from Red Sea coast meteorological data [20]; **defines the input uncertainty distribution.** |
| **Weibull scale parameter (Wind)** | c | 7.5 | m/s | Fitted from Red Sea coast meteorological data [20]; **defines the input uncertainty distribution.** |
| **Wind turbine cut-in speed** | vci | 3.0 | m/s | Manufacturer datasheet (2.9 MW turbine) |
| **Wind turbine rated speed** | vr | 12.0 | m/s | Manufacturer datasheet (2.9 MW turbine) |
| **Wind turbine cut-out speed** | vco | 25.0 | m/s | Manufacturer datasheet (2.9 MW turbine) |
| **PV temperature coefficient** | α | -0.0045 | 1/°C | Standard for crystalline silicon modules |
| **PV Nominal Operating Cell Temp.** | NOCT | 45 | °C | Manufacturer datasheet |
| **PV Standard Test Condition Irradiance** | G_STC | 1000 | W/m² | IEC 61215 standard |
| **PV Standard Test Condition Temp.** | T_STC | 25 | °C | IEC 61215 standard |
| **B. Power System & Operational Limits** | | | | |
| **Minimum voltage limit** | Vmin | 0.95 | p.u. | Egyptian Distribution Grid Code |
| **Maximum voltage limit** | Vmax | 1.05 | p.u. | Egyptian Distribution Grid Code |
| **Base load scaling factor (stress test)** | --- | 115 | % | Represents stressed Egyptian feeder conditions [5, 6] |
| **C. Economic Parameters** | | | | |
| **Capital Cost - PV System** | --- | 800 - 1000 | $/kW | Egyptian market estimates, 2024 |
| **Capital Cost - Wind System** | --- | 1200 - 1500 | $/kW | Egyptian market estimates, 2024 |
| **Capital Cost - D-STATCOM** | --- | 50 - 80 | $/kVAr | Industry estimates |
| **Capital Cost - Capacitor Bank** | --- | 15 - 25 | $/kVAr | Industry estimates |
| **Discount Rate (for LCOE)** | --- | 6.5 | % | Central Bank of Egypt benchmark |
| **Project Lifetime** | N | 20 | years | Standard for energy infrastructure |
| **D. Life-Cycle Assessment (LCA) Parameters** | | | | |
| **Emission Factor - PV Manufacturing** | EFM,PV | 80 | kg CO₂-eq/kW | Eco invent DB / Literature [23] |
| **Emission Factor - Wind Manufacturing** | EFM,WT | 40 | kg CO₂-eq/kW | Eco invent DB / Literature [23] |
| **Emission Factor - Grid Electricity (Egypt)** | EF grid | 0.55 | kg CO₂-eq/kWh | Egyptian Electricity Holding Co. report |
| **Recycled Fraction (Metals)** | R_REC | 0.85 | --- | Industry average for steel/aluminum |
| **E. Social & Policy Parameters** | | | | |
| **Employment Factor (Renewables)** | --- | 8.5 | FTE/MW | Derived from [22], contextualized for Egypt |
| **Social Weight - Job Creation** | α₁ | 0.7 | --- | Expert weighting, prioritizing local economic development |
| **Social Weight - Grid Independence** | α₂ | 0.3 | --- | Expert weighting, aligned with energy security goals |
| **F. Optimization Algorithm (NSGA-II)** | | | | |
| **Population Size** | Npop | 100 | individuals | Standard for problem scale; ensures diversity |
| **Maximum Generations** | Ngen | 200 | --- | Ensures convergence based on pilot runs |
| **Crossover Probability** | pc | 0.9 | --- | NSGA-II default / literature [12, 13] |
| **Mutation Probability** | P m | 1/n | --- | where *n* is number of decision variables |

**Table 2 : Complete LCA Parameters**

| **Life-Cycle Assessment (LCA) Parameters** | | | | |
| --- | --- | --- | --- | --- |
| **Manufacturing Stage** | | | | |
| **Emission Factor - PV Manufacturing** | **EF_M,PV** | **80** | **kg CO₂-eq/kW** | **Eco invent DB / Literature [23]** |
| **Emission Factor - Wind Turbine Manufacturing** | **EF_M,WT** | **40** | **kg CO₂-eq/kW** | **Eco invent DB / Literature [23]** |
| **Emission Factor - D-STATCOM Manufacturing** | **EF_M,DST** | **25** | **kg CO₂-eq/kVAr** | **Estimated from [23] for power electronics** |
| **Transportation Stage** | | | | |
| **Emission Factor - Road Transport** | **EFT,road** | **0.12** | **kg CO₂-eq/ton/km** | **Standard LCA database value** |
| **Average Transport Distance** | **Davg** | **300** | **km** | **Estimated Egypt-specific distance** |
| **Installation Stage** | | | | |
| **Emission Factor - Installation** | **EF_INS** | **5** | **kg CO₂-eq/kW** | **Construction emissions estimate [23]** |
| **Operation & Maintenance Stage** | | | | |
| **Emission Factor - Grid Electricity (Egypt)** | **EFgrid** | **0.55** | **kg CO₂-eq/kWh** | **Egyptian Electricity Holding Co. report** |
| **Annual O&M Energy Fraction** | **---** | **0.5** | **% of generation** | **Standard assumption for renewables** |
| **Decommissioning & Recycling Stage** | | | | |
| **Emission Factor - Decommissioning** | **EF_DEC** | **10** | **kg CO₂-eq/kW** | **Standard LCA value for dismantling** |
| **Recycled Fraction (Metals)** | **R_REC** | **0.85** | **---** | **Industry average for steel/aluminum** |
| **Avoided Emission Credit** | **---** | **-15** | **kg CO₂-eq/kW** | **Credit from recycling** |
