# Supplementary material for: A probabilistic multi objective decision framework for reactive power planning in der integrated microgrids enhancing sustainability and resilience in the egyptian grid
Source: Sci Rep. 2026 Aug 20;16:26167. doi: 10.1038/s41598-026-66944-w (PMC13494049; doi:10.1038/s41598-026-66944-w)
Supplement: Supplementary file 2 [file 41598_2026_66944_MOESM2_ESM.docx]

**S1 :Sensitivity Analysis**

To assess the robustness of scenario rankings under economic uncertainty, a comprehensive sensitivity analysis was performed considering key parameters that influence the economic viability of reactive power planning strategies.

**Sensitivity Analysis Parameters and Ranges**

| **Parameter** | **Baseline Value** | **Sensitivity Range** | **Rationale** |
| --- | --- | --- | --- |
| Technology Costs (CAPEX) | As per Table 3 | ±30% | Global price fluctuations [33] |
| Energy Price (Grid Tariff) | $0.142/kWh | ±20% | Subsidy reforms in Egypt [4,5] |
| Carbon Credit Value | $0/ton CO₂ | $25–75/ton CO₂ | Emerging carbon markets |
| Discount Rate | 10% | 5–15% | Financing uncertainty |
| Capital Subsidy | 0% | 0–40% | Egyptian incentive programs [18] |

**Sensitivity Analysis Results for Optimal Strategy (S5)**

| **Scenario** | **Payback Period (years)** | **IRR (%)** | **LCOE ($/kWh)** | **Ranking vs. Other Scenarios** |
| --- | --- | --- | --- | --- |
| Baseline | 9.2 | 10.1 | 0.091 | Optimal (S5) |
| Technology Costs -30% | 6.8 | 14.2 | 0.076 | Optimal (S5) |
| Technology Costs +30% | 11.5 | 7.8 | 0.108 | Optimal (S5) |
| Energy Price -20% | 10.8 | 8.5 | 0.095 | S2 competitive |
| Energy Price +20% | 7.9 | 12.3 | 0.087 | Optimal (S5) |
| Carbon Credit $50/ton | 8.4 | 11.8 | 0.086 | Optimal (S5) |
| Capital Subsidy 30% | 6.8 | 13.8 | 0.079 | Optimal (S5) |
| Combined Worst-Case | 12.4 | 6.5 | 0.118 | S2 marginally better |

S5 remains optimal when technical/social benefits included
Worst-case: +30% costs, -20% energy price, no carbon credits, 15% discount rate

**S2: Detailed System Parameters**

**IEEE 33-Bus Test System Parameters**

| Parameter | Value |
| --- | --- |
| Base Voltage | 12.66 kV |
| Base MVA | 100 MVA |
| Total Active Load | 3.715 MW |
| Total Reactive Load | 2.300 MVAr |
| Number of Buses | 33 |
| Number of Branches | 32 |
| Total Line Length | 38.8 km |

**Renewable Energy Resource Data (Red Sea Region, Egypt)**

| Parameter | Solar PV | Wind Turbine |
| --- | --- | --- |
| Average Resource | 5.8 kWh/m²/day | 7.2 m/s |
| Weibull Shape Parameter (k) | — | 2.1 |
| Weibull Scale Parameter (c) | — | 8.0 |
| Cut-in Speed (Vci) | — | 3 m/s |
| Rated Speed (Vr) | — | 12 m/s |
| Cut-out Speed (Vco) | — | 25 m/s |
| Temperature Coefficient (α) | -0.004/°C | — |
| NOCT | 45°C | — |

**S3: Economic Parameters**

**Cost Assumptions for DERs and Compensation Devices**

| Component | Capital Cost (CAPEX) | O&M Cost (% of CAPEX/year) | Lifetime (years) |
| --- | --- | --- | --- |
| PV System | $1,200/kW | 1.5% | 25 |
| Wind Turbine | $1,800/kW | 2.0% | 20 |
| Diesel Generator | $500/kW | 5.0% | 15 |
| Inverter | $300/kVA | 1.0% | 15 |
| Capacitor Bank | $40/kVAr | 0.5% | 15 |
| D-STATCOM | $80/kVAr | 1.0% | 20 |

**S4 :Life-Cycle Assessment Emission Factors**

| Stage | Emission Factor | Unit | Source |
| --- | --- | --- | --- |
| Manufacturing (PV) | 45 | kg CO₂/kW | [23] |
| Manufacturing (Wind) | 12 | kg CO₂/kW | [23] |
| Manufacturing (Inverter) | 8 | kg CO₂/kVA | [23] |
| Manufacturing (D-STATCOM) | 5 | kg CO₂/kVAr | [23] |
| Transportation | 0.05 | kg CO₂/ton-km | [23] |
| Installation | 2% of manufacturing | — | [23] |
| Operation (Grid) | 0.5 | kg CO₂/kWh | [23] |
| Decommissioning | 3% of manufacturing | — | [23] |
| Recycling Credit | -15% of manufacturing | — | [23] |

**S5 : Sensitivity Analysis Detailed Results**

**Complete Sensitivity Analysis Results for All Scenarios**

| Scenario | Parameter Variation | S1 NPV ($M) | S2 NPV ($M) | S3 NPV ($M) | S4 NPV ($M) | S5 NPV ($M) | Rank |
| --- | --- | --- | --- | --- | --- | --- | --- |
| Baseline | — | 4.2 | 5.1 | 5.8 | 5.3 | 6.2 | S5 |
| Tech Cost | -30% | 6.8 | 7.2 | 8.1 | 7.5 | 8.9 | S5 |
| Tech Cost | +30% | 2.1 | 3.3 | 3.6 | 3.2 | 3.8 | S5 |
| Energy Price | -20% | 3.1 | 4.2 | 4.5 | 4.0 | 4.7 | S5 |
| Energy Price | +20% | 5.4 | 6.0 | 7.2 | 6.5 | 7.8 | S5 |
| Carbon Credit | $25/ton | 4.8 | 5.3 | 6.1 | 5.6 | 6.5 | S5 |
| Carbon Credit | $50/ton | 5.5 | 5.6 | 6.4 | 5.9 | 6.8 | S5 |
| Carbon Credit | $75/ton | 6.2 | 5.8 | 6.7 | 6.2 | 7.1 | S5 |
| Discount Rate | 5% | 6.1 | 7.0 | 8.2 | 7.4 | 8.8 | S5 |
| Discount Rate | 15% | 2.8 | 3.7 | 4.0 | 3.8 | 4.3 | S5 |
| Subsidy | 10% | 4.8 | 5.5 | 6.3 | 5.8 | 6.8 | S5 |
| Subsidy | 20% | 5.4 | 5.9 | 6.8 | 6.3 | 7.4 | S5 |
| Subsidy | 30% | 6.0 | 6.3 | 7.3 | 6.8 | 8.0 | S5 |
| Subsidy | 40% | 6.6 | 6.7 | 7.8 | 7.3 | 8.6 | S5 |
| Worst-Case* | Combined | 1.2 | 2.1 | 1.9 | 1.8 | 2.0 | S2 |

*Worst-case: +30% costs, -20% energy price, no carbon credits, 15% discount rate, no subsidy

**S6 :Convergence Analysis**

**NSGA-II Convergence Statistics (10 Independent Runs)**

| Run | Generations to Converge | Final Hyper volume | Pareto Solutions |
| --- | --- | --- | --- |
| 1 | 195 | 0.871 | 48 |
| 2 | 188 | 0.873 | 52 |
| 3 | 203 | 0.868 | 47 |
| 4 | 192 | 0.872 | 51 |
| 5 | 197 | 0.870 | 49 |
| 6 | 185 | 0.874 | 53 |
| 7 | 202 | 0.869 | 48 |
| 8 | 191 | 0.872 | 50 |
| 9 | 198 | 0.871 | 49 |
| 10 | 194 | 0.873 | 51 |
| **Mean** | **194.5** | **0.871** | **49.8** |
| **Std Dev** | **5.8** | **0.002** | **2.0** |

**S7: Social Score Weighting Sensitivity**

**Impact of Social Weighting Factors on Scenario Ranking**

| α₁ (Jobs) | α₂ (Grid) | S1 Score | S2 Score | S3 Score | S4 Score | S5 Score | Optimal |
| --- | --- | --- | --- | --- | --- | --- | --- |
| 0.1 | 0.9 | 0.42 | 0.45 | 0.51 | 0.53 | 0.52 | S4 |
| 0.3 | 0.7 | 0.48 | 0.49 | 0.58 | 0.57 | 0.61 | S5 |
| 0.5 | 0.5 | 0.53 | 0.52 | 0.64 | 0.60 | 0.69 | S5 |
| 0.7 | 0.3 | 0.59 | 0.55 | 0.71 | 0.64 | 0.78 | S5 |
| 0.9 | 0.1 | 0.64 | 0.58 | 0.77 | 0.67 | 0.86 | S5 |

**S8 :Tornado Chart Data**

**Sensitivity of S5 NPV to Parameter Variations (± %)**

| Parameter | -30% | -20% | -10% | Baseline | +10% | +20% | +30% |
| --- | --- | --- | --- | --- | --- | --- | --- |
| Technology Cost | +22% | +15% | +7% | 0% | -8% | -15% | -22% |
| Energy Price | -15% | -10% | -5% | 0% | +5% | +10% | +12% |
| Carbon Credit | — | — | — | 0% | +4% | +8% | +12% |
| Discount Rate | +18% | +12% | +6% | 0% | -8% | -14% | -20% |
| Subsidy | +8% | +12% | +15% | 0% | — | — | — |
